# Diagnostic accuracy and added value of [18F]‐Florzolotau PET in the clinical workup of patients with cognitive symptoms

**DOI:** 10.1002/alz70856_102771

**Published:** 2025-12-24

**Authors:** Jin‐tai Yu, Jun Wang, Yu‐Yuan Huang

**Affiliations:** ^1^ National Center for Neurological Disorders, Shanghai, China; ^2^ Huashan hospital, Fudan University, Shanghai, China; ^3^ Daping Hospital, Third Military Medical University, Chongqing, China; ^4^ Department of Neurology and National Center for Neurological Disorders, Huashan Hospital, State Key Laboratory of Medical Neurobiology and MOE Frontiers Center, Fudan University, Shanghai, Shanghai, China

## Abstract

**Background:**

The novel positron emission tomography (PET) tracer [18F]Florzolotau allows in vivo detection of tau pathology and exhibits disease‐specific spatial pattern. We aimed to evaluate the diagnostic accuracy and added value of [18F]Florzolotau PET to a standard clinical workup in participants with cognitive symptoms.

**Method:**

In this real‐world cohort study, 1476 participants with congitive complaints who underwent a standard diagnostic workup, including medical history, clinical examination, neuropsychological assessments, routine blood tests, structural magnetic resonance imaging of brain and [18F]Florzolotau tau PET scan, were consecutively recruited at the tertiary memory clinics in Shanghai, China, from Jan 2019 through July 2024. Clinicians reported the diagnosis, diagnostic confidence and treatement plan before and after the tau PET reads, respectively. The primary outcomes were the diagnostic accuracy of tau PET pattern by visual read for tauopathies, and changes in diagnosis, diagnostic confidence and medication between the pre‐ and post‐PET reads. The secondary outcome was associations of variable factors with changes in diagnosis and diagnostic confidence.

**Result:**

A total of 1476 participants with a mean age at onset of 62.1 (SD, 9.6) years (775 female) were included. Based on disease‐specific spatial pattern, [18F]Florzolotau PET exhibited the overall accuracy of 97.2%, 95.3%, 94.7% and 97.7%, respectively, for separating Alzheimer's disease (AD), frontotemporal dementia (FTD), progressive supranuclear palsy (PSP) and corticobasal syndrome (CBS) from others. The tau PET results led to a change in diagnoses in 318 participants (21.5%), an increase of diagnostic confidence from 67.7% to 80.7%, and a change in medication in 405 participants (27.4%). Cognitive impairment at the MCI and dementia level, co‐existing motor systems and a lower baseline diagnostic confidence (<70%) were associated with the changes of diagnosis and diagnostic confidence.

**Conclusion:**

[18F]Florzolotau tau PET has high accuracy in separating cognitive disorders, and exhibits added value on the routine clinical workup in patients with cognitive symptoms. The added value is more pronounced in MCI or dementia participants with lower diagnostic confidence and without co‐existing motor symptoms, suggesting the application of [18F]Florzolotau tau PET in appropriate patients in memory clinics.

